# Supplementary material for: Comparative Transcriptome Analysis Reveals the Transcriptional Alterations in Growth- and Development-Related Genes in Sweet Potato Plants Infected and Non-Infected by SPFMV, SPV2, and SPVG
Source: Int J Mol Sci. 2019 Feb 26;20(5):1012. doi: 10.3390/ijms20051012 (PMC6429102; doi:10.3390/ijms20051012)
Supplement: Supplementary file 1 [file ijms-20-01012-s001.zip › Supplementary Files/Table 5S.doc]

Table 5S KEGG Pathway enrichment analysis based on the differentially up-regulated genes between VFSP and VCSP leaves

| KEGG Pathway | Number of genes | -log_10_(P value)* |
| --- | --- | --- |
| Photosynthesis - antenna proteins (ko00196) | 25 | 12.08 |
| Photosynthesis (ko00195) | 20 | 7.12 |
| Porphyrin and chlorophyll metabolism (ko00860) | 19 | 3.57 |
| Carbon fixation in photosynthetic organisms (ko00710) | 20 | 3.32 |
| Nitrogen metabolism (ko00910) | 14 | 3.13 |
| Steroid biosynthesis (ko00100) | 10 | 1.96 |
| Glyoxylate and dicarboxylate metabolism (ko00630) | 18 | 1.79 |
| Ubiquitin mediated proteolysis (ko04120) | 25 | 1.67 |
| Phosphonate and phosphinate metabolism (ko00440) | 4 | 1.61 |
| N-Glycan biosynthesis (ko00510) | 10 | 1.47 |

*P values of all GO terms are lower than 0.05. Conversely, −log 10 (P value) values of all GO terms are greater than 1.3010, that is, the greater -log 10(P value) value, the better significance.
