# Supplementary material for: Comparative Transcriptome Analysis Reveals the Transcriptional Alterations in Growth- and Development-Related Genes in Sweet Potato Plants Infected and Non-Infected by SPFMV, SPV2, and SPVG
Source: Int J Mol Sci. 2019 Feb 26;20(5):1012. doi: 10.3390/ijms20051012 (PMC6429102; doi:10.3390/ijms20051012)
Supplement: Supplementary file 1 [file ijms-20-01012-s001.zip › Supplementary Files/Table 6S.doc]

Table 6S KEGG Pathway enrichment analysis based on the differentially up-regulated genes between of VFSP and VCSP storage roots

| KEGG Pathway | Number of genes | -log_10_(P value)* |
| --- | --- | --- |
| Linoleic acid metabolism (ko00591) | 5 | 4.37 |
| Thiamine metabolism (ko00730) | 5 | 4.29 |
| Monobactam biosynthesis (ko00261) | 5 | 4.21 |
| Selenocompound metabolism (ko00450) | 5 | 3.92 |
| Sulfur metabolism (ko00920) | 5 | 3.56 |
| Thyroid hormone synthesis (ko04918) | 4 | 3.13 |
| Glyoxylate and dicarboxylate metabolism (ko00630) | 7 | 2.92 |
| Ribosome biogenesis in eukaryotes (ko03008) | 7 | 2.47 |
| Chlorocyclohexane and chlorobenzene degradation (ko00361) | 2 | 2.21 |
| Toluene degradation (ko00623) | 2 | 2.21 |
| Fluorobenzoate degradation (ko00364) | 2 | 2.21 |
| Circadian rhythm - plant (ko04712) | 4 | 1.91 |
| Prion diseases (ko05020) | 2 | 1.61 |
| NOD-like receptor signaling pathway (ko04621) | 2 | 1.47 |
| Purine metabolism (ko00230) | 7 | 1.47 |
| Nitrogen metabolism (ko00910) | 3 | 1.40 |
| Folate biosynthesis (ko00790) | 2 | 1.39 |
| Tropane, piperidine and pyridine alkaloid biosynthesis (ko00960) | 3 | 1.38 |
| Sesquiterpenoid and triterpenoid biosynthesis （ko00909） | 3 | 1.32 |

*P values of all GO terms are lower than 0.05. Conversely, −log 10 (P value) values of all GO terms are greater than 1.3010, that is, the greater -log 10(P value) value, the better significance.
