# Supplementary material for: Comparative Transcriptome Analysis Reveals the Transcriptional Alterations in Growth- and Development-Related Genes in Sweet Potato Plants Infected and Non-Infected by SPFMV, SPV2, and SPVG
Source: Int J Mol Sci. 2019 Feb 26;20(5):1012. doi: 10.3390/ijms20051012 (PMC6429102; doi:10.3390/ijms20051012)
Supplement: Supplementary file 1 [file ijms-20-01012-s001.zip › Supplementary Files/Table 7S.doc]

Table 7S KEGG Pathway enrichment analysis based on the differentially down-regulated genes between VCSP and VFSP leaves

| KEGG Pathway | Number of genes | -log_10_(P value)* |
| --- | --- | --- |
| Ribosome (ko03010) | 180 | 18.21 |
| Flavonoid biosynthesis (ko00941) | 28 | 4.30 |
| Phenylalanine metabolism (ko00360) | 28 | 2.67 |
| Stilbenoid, diarylheptanoid and gingerol biosynthesis (ko00945) | 16 | 2.64 |
| Biosynthesis of amino acids (ko01230) | 95 | 2.59 |
| Plant hormone signal transduction (ko04075) | 73 | 2.39 |
| Protein processing in endoplasmic reticulum (ko04141) | 73 | 2.25 |
| alpha-Linolenic acid metabolism (ko00592) | 29 | 1.92 |
| Biosynthesis of unsaturated fatty acids (ko01040) | 16 | 1.85 |
| Phenylpropanoid biosynthesis (ko00940) | 61 | 1.60 |
| Glutathione metabolism (ko00480) | 35 | 1.51 |
| Phenylalanine, tyrosine and tryptophan biosynthesis (ko00400) | 22 | 1.51 |
| Linoleic acid metabolism (ko00591) | 10 | 1.46 |
| Fatty acid elongation (ko00062) | 9 | 1.44 |
| Galactose metabolism (ko00052) | 23 | 1.34 |

*P values of all GO terms are lower than 0.05. Conversely, −log 10 (P value) values of all GO terms are greater than 1.3010, that is, the greater -log 10(P value) value, the better significance.
