# Supplementary material for: Comparative Transcriptome Analysis Reveals the Transcriptional Alterations in Growth- and Development-Related Genes in Sweet Potato Plants Infected and Non-Infected by SPFMV, SPV2, and SPVG
Source: Int J Mol Sci. 2019 Feb 26;20(5):1012. doi: 10.3390/ijms20051012 (PMC6429102; doi:10.3390/ijms20051012)
Supplement: Supplementary file 1 [file ijms-20-01012-s001.zip › Supplementary Files/Table 8S.doc]

Table 8S KEGG Pathway enrichment analysis based on the differentially down-regulated genes between VCSP and VFSP storage roots

| KEGG Pathway | Number of genes | -log_10_(P value)* |
| --- | --- | --- |
| Alanine, aspartate and glutamate metabolism (ko00250) | 9 | 4.27 |
| Monoterpenoid biosynthesis (ko00902) | 5 | 4.16 |
| Galactose metabolism (ko00052) | 7 | 3.27 |
| GABAergic synapse (ko04727) | 6 | 3.27 |
| Two-component system (ko02020) | 4 | 2.96 |
| Cysteine and methionine metabolism (ko00270) | 9 | 2.79 |
| Antigen processing and presentation (ko04612) | 5 | 2.67 |
| Glutamatergic synapse (ko04724) | 5 | 2.60 |
| Basal cell carcinoma (ko05217) | 2 | 2.05 |
| Apoptosis (ko04210) | 3 | 1.96 |
| Nitrogen metabolism (ko00910) | 4 | 1.86 |
| Gap junction (ko04540) | 3 | 1.83 |
| Arginine biosynthesis (ko00220) | 4 | 1.83 |
| Plant hormone signal transduction (ko04075) | 10 | 1.76 |
| Pathogenic Escherichia coli infection (ko05130) | 3 | 1.57 |
| Glyoxylate and dicarboxylate metabolism (ko00630) | 5 | 1.36 |
| Porphyrin and chlorophyll metabolism (ko00860) | 4 | 1.32 |
| Taurine and hypotaurine metabolism (ko00430) | 2 | 1.32 |
| Osteoclast differentiation (ko04380) | 2 | 1.32 |

*P values of all GO terms are lower than 0.05. Conversely, −log 10 (P value) values of all GO terms are greater than 1.3010, that is, the greater -log 10(P value) value, the better significance.
