# Supplementary material for: Comparative Transcriptome Analysis Reveals the Transcriptional Alterations in Growth- and Development-Related Genes in Sweet Potato Plants Infected and Non-Infected by SPFMV, SPV2, and SPVG
Source: Int J Mol Sci. 2019 Feb 26;20(5):1012. doi: 10.3390/ijms20051012 (PMC6429102; doi:10.3390/ijms20051012)
Supplement: Supplementary file 1 [file ijms-20-01012-s001.zip › Supplementary Files/Table S1.doc]

Additional file 1 Overall GO classification of differentially up-regulated genes of VFSP/VCSP leaves

| Gene Ontology term | Number of genes | -log_10_(P value)* |
| --- | --- | --- |
| **Cell component** |  |  |
| photosystem (GO:0009521) | 21 | 13.85 |
| photosystem I (GO:0009522) | 14 | 11.51 |
| chloroplast thylakoid (GO:0009534) | 40 | 10.96 |
| thylakoid (GO:0009579) | 40 | 10.96 |
| plastid thylakoid (GO:0031976) | 40 | 10.96 |
| photosynthetic membrane (GO:0034357) | 32 | 10.60 |
| thylakoid part (GO:0044436) | 37 | 10.18 |
| chloroplast thylakoid membrane (GO:0009535) | 31 | 10.05 |
| thylakoid membrane (GO:0042651) | 31 | 10.05 |
| plastid thylakoid membrane (GO:0055035) | 31 | 10.05 |
| organelle subcompartment (GO:0031984) | 42 | 9.66 |
| photosystem II (GO:0009523) | 15 | 9.12 |
| membrane protein complex (GO:0098796) | 23 | 8.92 |
| chloroplast part (GO:0044434) | 73 | 8.89 |
| plastid part (GO:0044435) | 73 | 8.66 |
| chloroplast (GO:0009507) | 123 | 7.77 |
| plastid (GO:0009536) | 123 | 7.59 |
| cytoplasmic part (GO:0044444) | 248 | 7.34 |
| membrane (GO:0016020) | 219 | 5.28 |
| intracellular organelle part (GO:0044446) | 167 | 4.30 |
| organelle part (GO:0044422) | 167 | 4.20 |
| chloroplast stroma (GO:0009570) | 26 | 3.57 |
| membrane part (GO:0044425) | 176 | 3.49 |
| plastid stroma (GO:0009532) | 26 | 3.42 |
| cytoplasm (GO:0005737) | 279 | 3.30 |
| extracellular organelle (GO:0043230) | 4 | 2.94 |
| extracellular exosome (GO:0070062) | 4 | 2.94 |
| extracellular vesicle (GO:1903561) | 4 | 2.94 |
| protein complex (GO:0043234) | 32 | 2.88 |
| intrinsic component of membrane (GO:0031224) | 164 | 2.70 |
| integral component of membrane (GO:0016021) | 158 | 2.43 |
| extracellular region part (GO:0044421) | 7 | 2.19 |
| nuclear periphery (GO:0034399) | 3 | 2.13 |
| tethering complex (GO:0099023) | 3 | 2.13 |
| Golgi apparatus part (GO:0044431) | 29 | 2.10 |
| Golgi membrane (GO:0000139) | 28 | 2.00 |
| bounding membrane of organelle (GO:0098588) | 50 | 1.80 |
| membrane-bounded organelle (GO:0043227) | 298 | 1.70 |
| Golgi apparatus (GO:0005794) | 34 | 1.62 |
| intracellular membrane-bounded organelle (GO:0043231) | 291 | 1.53 |
| organelle membrane (GO:0031090) | 70 | 1.46 |
| endomembrane system (GO:0012505) | 66 | 1.42 |
| chromosomal region (GO:0098687) | 4 | 1.39 |
| GARP complex (GO:0000938) | 2 | 1.36 |
| nuclear lamina (GO:0005652) | 2 | 1.36 |
| vacuolar membrane (GO:0005774) | 15 | 1.33 |
| Golgi-associated vesicle （GO:0005798） | 3 | 1.32 |
| cell cortex （GO:0005938） | 3 | 1.32 |
| COPI-coated vesicle （GO:0030137） | 3 | 1.32 |
| Golgi-associated vesicle membrane （GO:0030660） | 3 | 1.32 |
| COPI-coated vesicle membrane （GO:0030663） | 3 | 1.32 |
| cytoplasmic region （GO:0099568） | 3 | 1.32 |
| **Molecular function** |  |  |
| chlorophyll binding （GO:0016168） | 11 | 9.00 |
| tetrapyrrole binding （GO:0046906） | 11 | 9.00 |
| carbon-oxygen lyase activity, acting on polysaccharides （GO:0016837） | 4 | 2.96 |
| pectate lyase activity （GO:0030570） | 4 | 2.96 |
| catalytic activity （GO:0003824） | 304 | 2.77 |
| oxidoreductase activity, acting on other nitrogenous compounds as donors （GO:0016661） | 3 | 2.72 |
| magnesium chelatase activity （GO:0016851） | 3 | 2.72 |
| ligase activity, forming nitrogen-metal bonds （GO:0051002） | 3 | 2.72 |
| ligase activity, forming nitrogen-metal bonds, forming coordination complexes （GO:0051003） | 3 | 2.72 |
| histone acetyltransferase activity （GO:0004402） | 4 | 2.22 |
| peptide N-acetyltransferase activity （GO:0034212） | 4 | 2.22 |
| peptide-lysine-N-acetyltransferase activity （GO:0061733） | 4 | 2.22 |
| protein kinase binding （GO:0019901） | 5 | 2.15 |
| carboxypeptidase activity （GO:0004180） | 6 | 1.88 |
| oxidoreductase activity （GO:0016491） | 67 | 1.83 |
| intramolecular transferase activity （GO:0016866） | 5 | 1.80 |
| N-acetyltransferase activity （GO:0008080） | 4 | 1.75 |
| kinase binding （GO:0019900） | 5 | 1.66 |
| active transmembrane transporter activity （GO:0022804） | 18 | 1.60 |
| N-acyltransferase activity （GO:0016410） | 4 | 1.58 |
| oxidosqualene cyclase activity（GO:0031559） | 3 | 1.55 |
| protein histidine kinase binding （GO:0043424） | 3 | 1.55 |
| carbon-oxygen lyase activity （GO:0016835） | 10 | 1.54 |
| Actin binding （GO:0003779） | 7 | 1.46 |
| antiporter activity （GO:0015297） | 7 | 1.46 |
| exopeptidase activity （GO:0008238） | 7 | 1.38 |
| 1,4-alpha-glucan branching enzyme activity （GO:0003844） | 2 | 1.37 |
| glucose-6-phosphate dehydrogenase activity （GO:0004345） | 2 | 1.37 |
| hydroxyacylglutathione hydrolase activity （GO:0004416） | 2 | 1.37 |
| phosphatidylinositol-4,5-bisphosphate 5-phosphatase activity （GO:0004439） | 2 | 1.37 |
| sulfate adenylyltransferase activity （GO:0004779） | 2 | 1.37 |
| glycerophosphodiester phosphodiesterase activity （GO:0008889） | 2 | 1.37 |
| oxidoreductase activity, acting on paired donors, with oxidation of a pair of donors resulting in the reduction of molecular oxygen to two molecules of water （GO:0016717） | 2 | 1.37 |
| phosphatidylinositol bisphosphate phosphatase activity （GO:0034593） | 2 | 1.37 |
| phosphatidylinositol trisphosphate phosphatase activity （GO:0034594） | 2 | 1.37 |
| phosphatidylinositol phosphate 5-phosphatase activity (GO:0034595) | 2 | 1.37 |
| fructose 1,6-bisphosphate 1-phosphatase activity (GO:0042132) | 2 | 1.37 |
| glyceraldehyde-3-phosphate dehydrogenase (NADP+) (phosphorylating) activity (GO:0047100) | 2 | 1.37 |
| carbohydrate derivative transporter activity (GO:1901505) | 3 | 1.34 |
| monooxygenase activity (GO:0004497) | 12 | 1.31 |
| **Biological process** |  |  |
| photosynthesis (GO:0015979) | 38 | 18.37 |
| protein-chromophore linkage (GO:0018298) | 12 | 5.96 |
| carbohydrate biosynthetic process (GO:0016051) | 26 | 5.05 |
| single-organism carbohydrate metabolic process (GO:0044723) | 34 | 3.64 |
| polysaccharide biosynthetic process (GO:0000271) | 16 | 3.59 |
| polysaccharide metabolic process (GO:0005976) | 27 | 3.54 |
| reductive pentose-phosphate cycle (GO:0019253) | 7 | 3.54 |
| photosynthesis, dark reaction (GO:0019685) | 7 | 3.54 |
| porphyrin-containing compound biosynthetic process(GO:0006779) | 10 | 3.29 |
| tetrapyrrole biosynthetic process (GO:0033014) | 10 | 3.29 |
| transition metal ion homeostasis (GO:0055076) | 7 | 3.25 |
| photosynthesis, light reaction (GO:0019684) | 6 | 3.17 |
| carbon fixation (GO:0015977) | 8 | 3.15 |
| carbohydrate metabolic process (GO:0005975) | 46 | 3.12 |
| cellular carbohydrate metabolic process (GO:0044262) | 20 | 2.99 |
| porphyrin-containing compound metabolic process (GO:0006778) | 10 | 2.97 |
| tetrapyrrole metabolic process (GO:0033013) | 10 | 2.97 |
| chlorophyll biosynthetic process (GO:0015995) | 9 | 2.94 |
| metal ion homeostasis (GO:0055065) | 7 | 2.78 |
| cation homeostasis (GO:0055080) | 7 | 2.78 |
| photosynthesis, light harvesting (GO:0009765) | 3 | 2.64 |
| chlorophyll metabolic process (GO:0015994) | 9 | 2.63 |
| inorganic ion homeostasis (GO:0098771) | 7 | 2.58 |
| iron ion homeostasis (GO:0055072) | 5 | 2.50 |
| pigment biosynthetic process (GO:0046148) | 12 | 2.49 |
| cellular carbohydrate biosynthetic process (GO:0034637) | 12 | 2.39 |
| pectin metabolic process (GO:0045488) | 11 | 2.29 |
| ion homeostasis (GO:0050801) | 7 | 2.24 |
| cellular polysaccharide metabolic process (GO:0044264) | 13 | 2.21 |
| galacturonan metabolic process (GO:0010393) | 11 | 2.19 |
| single-organism biosynthetic process (GO:0044711) | 70 | 2.18 |
| cellular glucan metabolic process (GO:0006073) | 12 | 2.11 |
| pigment metabolic process (GO:0042440) | 12 | 2.11 |
| glucan metabolic process (GO:0044042) | 12 | 2.11 |
| cellular polysaccharide biosynthetic process (GO:0033692) | 10 | 2.10 |
| sulfate transport (GO:0008272) | 3 | 2.08 |
| sulfur compound transport (GO:0072348) | 3 | 2.08 |
| macromolecule modification (GO:0043412) | 51 | 2.06 |
| pectin biosynthetic process (GO:0045489) | 6 | 2.05 |
| terpenoid metabolic process (GO:0006721) | 12 | 2.02 |
| glucan biosynthetic process (GO:0009250) | 9 | 2.01 |
| starch biosynthetic process (GO:0019252) | 6 | 1.89 |
| response to high light intensity (GO:0009644) | 4 | 1.87 |
| lipid modification (GO:0030258) | 4 | 1.87 |
| terpenoid biosynthetic process (GO:0016114) | 11 | 1.76 |
| starch metabolic process (GO:0005982) | 6 | 1.75 |
| regulation of biological quality (GO:0065008) | 16 | 1.75 |
| photosynthetic electron transport chain (GO:0009767) | 3 | 1.73 |
| electron transport chain (GO:0022900) | 3 | 1.73 |
| cellular transition metal ion homeostasis (GO:0046916) | 3 | 1.73 |
| phototransduction (GO:0007602) | 5 | 1.69 |
| detection of external stimulus (GO:0009581) | 5 | 1.69 |
| detection of abiotic stimulus (GO:0009582) | 5 | 1.69 |
| detection of light stimulus (GO:0009583) | 5 | 1.69 |
| red, far-red light phototransduction (GO:0009585) | 5 | 1.69 |
| red or far-red light signaling pathway (GO:0010017) | 5 | 1.69 |
| cellular response to nutrient levels (GO:0031669) | 5 | 1.69 |
| cellular response to red or far red light (GO:0071489) | 5 | 1.69 |
| regionalization (GO:0003002) | 4 | 1.66 |
| exocytosis (GO:0006887) | 4 | 1.66 |
| chemical homeostasis (GO:0048878) | 7 | 1.61 |
| isoprenoid metabolic process (GO:0006720) | 14 | 1.61 |
| detection of stimulus (GO:0051606) | 5 | 1.55 |
| cellular protein modification process (GO:0006464) | 38 | 1.49 |
| protein modification process (GO:0036211) | 38 | 1.49 |
| pattern specification process (GO:0007389) | 4 | 1.48 |
| regulation of cellular component size (GO:0032535) | 4 | 1.48 |
| secretion by cell (GO:0032940) | 4 | 1.48 |
| secretion (GO:0046903) | 4 | 1.48 |
| regulation of anatomical structure size (GO:0090066) | 4 | 1.48 |
| cellular ion homeostasis (GO:0006873) | 3 | 1.47 |
| cellular metal ion homeostasis (GO:0006875) | 3 | 1.47 |
| chromosome segregation (GO:0007059) | 3 | 1.47 |
| cellular cation homeostasis (GO:0030003) | 3 | 1.47 |
| glyceraldehyde-3-phosphate metabolic process (GO:0019682) | 5 | 1.42 |
| response to nutrient levels (GO:0031667) | 5 | 1.42 |
| cell communication (GO:0007154) | 35 | 1.41 |
| oxidation-reduction process (GO:0055114) | 74 | 1.40 |
| isoprenoid biosynthetic process (GO:0008299) | 13 | 1.39 |
| single-organism transport (GO:0044765) | 26 | 1.35 |
| single-organism localization (GO:1902578) | 26 | 1.35 |
| cofactor biosynthetic process (GO:0051188) | 13 | 1.34 |
| cellular iron ion homeostasis (GO:0006879) | 2 | 1.32 |
| response to low light intensity stimulus (GO:0009645) | 2 | 1.32 |
| dephosphorylation (GO:0016311) | 2 | 1.32 |
| carbohydrate transmembrane transport (GO:0034219) | 2 | 1.32 |
| regulation of DNA methylation (GO:0044030) | 2 | 1.32 |
| alcohol catabolic process (GO:0046164) | 2 | 1.32 |
| protein modification by small protein removal (GO:0070646) | 2 | 1.32 |
| regulation of nucleus size (GO:0097298) | 2 | 1.32 |
| response to paraquat (GO:1901562) | 2 | 1.32 |
| response to red or far red light (GO:0009639) | 6 | 1.31 |
| cellular response to extracellular stimulus (GO:0031668) | 5 | 1.30 |

*P values of all GO terms are lower than 0.05. Conversely, −log 10 (P value) values of all GO terms are greater than 1.3010, that is, the greater -log 10(P value) value, the better significance.
