# Supplementary material for: Comparative Transcriptome Analysis Reveals the Transcriptional Alterations in Growth- and Development-Related Genes in Sweet Potato Plants Infected and Non-Infected by SPFMV, SPV2, and SPVG
Source: Int J Mol Sci. 2019 Feb 26;20(5):1012. doi: 10.3390/ijms20051012 (PMC6429102; doi:10.3390/ijms20051012)
Supplement: Supplementary file 1 [file ijms-20-01012-s001.zip › Supplementary Files/Table S10.doc]

**Table S10** Thirty pairs of qPCR primers

| **No.** | **Forward primer** | | **Reverse primer** | **Functional classification of genes** |
| --- | --- | --- | --- | --- |
| **3 pairs of qPCR Primers for 3 virus genes-specific transcript amplification** | | | |  |
| 1 | SPFMW-F: 5’-CATGCCAAGGTATGGTCTACAACG-3’ | SPFMW-R: 5’- GCTGCTTTCATCTGTAAATGTGCTTC-3’ | |  |
| 2 | SPV2-F: 5’-CACCAGAAGGGAGCACGAATC-3’ | SPV2-R: 5’- GTCCTGTATCTCGCCCATAAGTGT-3’ | |  |
| 3 | SPVG-F: 5’- GACCACTTGACAATCTATGACCCTG-3’ | SPVG-R: 5’-CCCCATTTGCTCATCATTCACTCCA-3’ | |  |
| **26 pairs of qPCR Primers for 26 differentially expressed genes-specific transcript amplification** | | | |  |
| 4 | G23362-F: 5’- CTTGGGCAACCCGAACCTGAT -3’ | G23362-R: 5’- CCCACTCTGTATCCCTCAACGA -3’ | | Photosynthesis - antenna proteins |
| 5 | G11966-F: 5’- CATCAAGCCTGGATGCGTGAAC -3’ | G11966-R: 5’- CCGAGTGGGTCGAACCATAAGC -3’ | | Photosynthesis - antenna proteins |
| 6 | G10803-F: 5’- CAGATTCAGAGGGAGGATTTGACC -3’ | G10803-R: 5’- CTCCGACCACTGGAGTTGATGT -3’ | | Photosynthesis |
| 7 | G5733-F: 5’- CTAACCAGTGTCCCACCATTGAA -3’ | G5733-R: 5’- GAGGTTGGCTCCAGGCAGAA -3’ | | Photosynthesis |
| 8 | G21268-F: 5’- CTGAATCGTCTCACGCCACTG -3’ | G21268-R: 5’- GCAGCAACTCTATCCTCGCTTCTA -3’ | | Porphyrin and chlorophyll metabolism |
| 9 | G14549-F: 5’- GATAGGGTATGCGGGACAATCG -3’ | G14549-R: 5’- CATCCACTAAATGGTCATCCAACAG -3’ | | Porphyrin and chlorophyll metabolism |
| 10 | G14505-F: 5’- GGATGCTGTGACTACGCACTTG -3’ | G14505-R: 5’- GCCACTCCTGCCTACGCTCTT -3’ | | Carbon fixation in photosynthetic organisms |
| 11 | G23115-F: 5’- GCCAAGCGTACACCCTAAAGC -3’ | G23115-R: 5’- GCTAGTGGGATTAAGCATCACCAG -3’ | | Carbon fixation in photosynthetic organisms |
| 12 | G34812-F: 5’- GCAACTACATTGGCGGAACCTT -3’ | G34812-R: 5’- GTACCACGGCATAGCCTTCATT -3’ | | Steroid biosynthesis |
| 13 | G20348-F: 5’- CATTCAGTATGAAGATGAAGCCACT -3’ | G20348-R: 5’- GTCCTCTATCCAACGACAAAGC -3’ | | Steroid biosynthesis |
| 14 | G5606-F: 5’- GTGAGGAAGGACGACGAGGTT -3’ | G5606-R: 5’- GATGCCGACGTTGACAGTGGAT -3’ | | Ribosome |
| 15 | G36166-F: 5’- CCACGGGAGGAAGTGAAGACA -3’ | G36166-R: 5’- CCCTGCAAGGTTACTGGAAAG -3’ | | Ribosome |
| 16 | G28034-F: 5’- CCCATTCATCACCCCAACATTC -3’ | G28034-R: 5’- CAGATGCACATACTGCCCTTGC -3’ | | Flavonoid biosynthesis |
| 17 | G37782-F: 5’- GGGTTCTGTTTGTGGAGGCTGAG -3’ | G37782-R: 5’- GGGTTACCTGGAAAATGATGAGTG -3’ | | Flavonoid biosynthesis |
| 18 | G2984-F: 5’- CTTGGACAGTTCATTCTCCCAGAG -3’ | G2984-R: 5’- - CTCGTAGAGGGCATTCACATCG 3’ | | Linoleic acid metabolism |
| 19 | G43630-F: 5’- GGTTGAGGGATGATGAGTTTGC -3’ | G43630-R: 5’- GTGATTGCTGATTCAGGAGGAC -3’ | | Linoleic acid metabolism |
| 20 | G23858-F: 5’- GTACGACGAGCAAGACAACTACG -3’ | G23858-R: 5’- CGCCCTTCACTATCAAATCCT -3’ | | Thiamine metabolism |
| 21 | G33925-F: 5’- GATGGATGGACCAGTTTGCTTTG -3’ | G33925-R: 5’- GCACCTTCAGACGGTAGAGTTTC -3’ | | Thiamine metabolism |
| 22 | G8518-F: 5’- CCTGGACTGGAGCGGCTAAAC -3’ | G8518-R: 5’- CCGGTGGGTTCTCCTTGTTCT -3’ | | Monobactam biosynthesis |
| 23 | G9518-F: 5’- GGTGCCAATGTCTTGCATCCT -3’ | G9518-R: 5’- CTCCATCTTCAAACTCGGTCTCAT -3’ | | Monobactam biosynthesis |
| 24 | G10497-F: 5’- GATCATATCTCAAAGAGCGTCTCG -3’ | G10497-R: 5’- GGCCTCGTCCCCTTACATCG -3’ | | Alanine, aspartate and glutamate metabolism |
| 25 | G44676-F: 5’- CCTGGACCACAGGGACCATACT -3’ | G44676-R: 5’- CCGGGCATCACTTCTCCGTTA -3’ | | Alanine, aspartate and glutamate metabolism |
| 26 | G27213- F: 5’- CACCTGAAAAGCTGGATGAAGTG -3’ | G27213-R: 5’- CATAGGCTGACATGCGTGGAG -3’ | | Monoterpenoid biosynthesis |
| 27 | G38822-F: 5’- GAAGCATTTCTTCCCCTCCTCC -3’ | G38822-R: 5’- GGCCCACTCATCGCATATTTT -3’ | | Monoterpenoid biosynthesis |
| 28 | G20899-F: 5’- GATGGCACAGGGGTTCGTGAT -3’ | G20899-R: 5’- CCTATTGAAGGGTCTGCAAGTTTCT -3’ | | Galactose metabolism |
| 29 | G26285-F: 5’- GACCATAACCATCCCAGTTCCAG -3’ | G26285-R: 5’- GAAGCCTCACTCGCCTTCATCC -3’ | | Galactose metabolism |
| **One pairs of qPCR Primers for an internal standard gene-specific transcript amplification** | | | |  |
| 30 | ARF – F: 5’-GCTCTTTGCCAAGAAGGAGATGC-3’ | ARF – R: 5’-CGAATCTTGTCCTGACCACCAACA-3’ | |  |
