# Supplementary material for: Comparative Transcriptome Analysis Reveals the Transcriptional Alterations in Growth- and Development-Related Genes in Sweet Potato Plants Infected and Non-Infected by SPFMV, SPV2, and SPVG
Source: Int J Mol Sci. 2019 Feb 26;20(5):1012. doi: 10.3390/ijms20051012 (PMC6429102; doi:10.3390/ijms20051012)
Supplement: Supplementary file 1 [file ijms-20-01012-s001.zip › Supplementary Files/Table S2.doc]

Table S2 Overall GO classification of differentially up-regulated genes of VFSP/VCSP storage roots

| Gene ontology term | Number of genes | -log_10_(P value)* |
| --- | --- | --- |
| **Cell component** |  |  |
| anchored component of membrane (GO:0031225) | 3 | 1.77 |
| nucleolus (GO:0005730) | 3 | 1.51 |
| endoplasmic reticulum lumen (GO:0005788) | 2 | 1.41 |
| **Molecular function** |  |  |
| 4 iron, 4 sulfur cluster binding (GO:0051539) | 4 | 3.15 |
| oxidoreductase activity, acting on other nitrogenous compounds as donors (GO:0016661) | 2 | 3.09 |
| iron-sulfur cluster binding (GO:0051536) | 4 | 2.30 |
| metal cluster binding (GO:0051540) | 4 | 2.30 |
| xyloglucan:xyloglucosyl transferase activity (GO:0016762) | 2 | 1.65 |
| oxidoreductase activity, acting on the CH-NH2 group of donors (GO:0016638) | 2 | 1.60 |
| signaling receptor activity (GO:0038023) | 2 | 1.45 |
| ATPase activity (GO:0016887) | 3 | 1.36 |
| GTP cyclohydrolase activity (GO:0003933) | 1 | 1.30 |
| sulfate adenylyltransferase activity (GO:0004779) | 1 | 1.30 |
| phosphotransferase activity, paired acceptors (GO:0016781) | 1 | 1.30 |
| amino acid kinase activity (GO:0019202) | 1 | 1.30 |
| **Biological process** |  |  |
| sulfur compound metabolic process (GO:0006790) | 6 | 3.60 |
| sulfur compound biosynthetic process (GO:0044272) | 5 | 3.29 |
| organonitrogen compound biosynthetic process (GO:1901566) | 12 | 2.76 |
| thiamine metabolic process (GO:0006772) | 2 | 2.58 |
| thiamine biosynthetic process (GO:0009228) | 2 | 2.58 |
| thiamine-containing compound metabolic process (GO:0042723) | 2 | 2.58 |
| thiamine-containing compound biosynthetic process (GO:0042724) | 2 | 2.58 |
| nitrate metabolic process (GO:0042126) | 3 | 2.52 |
| nitrate assimilation (GO:0042128) | 3 | 2.52 |
| reactive nitrogen species metabolic process (GO:2001057) | 3 | 2.52 |
| water-soluble vitamin metabolic process (GO:0006767) | 3 | 2.39 |
| water-soluble vitamin biosynthetic process (GO:0042364) | 3 | 2.39 |
| cellular amide metabolic process (GO:0043603) | 7 | 2.29 |
| nitrogen cycle metabolic process (GO:0071941) | 3 | 2.27 |
| organonitrogen compound metabolic process (GO:1901564) | 13 | 2.20 |
| peptide metabolic process (GO:0006518) | 6 | 2.09 |
| vitamin metabolic process (GO:0006766) | 3 | 2.06 |
| vitamin biosynthetic process (GO:0009110) | 3 | 2.06 |
| plant organ formation (GO:1905393) | 2 | 2.04 |
| ribosome biogenesis (GO:0042254) | 3 | 2.01 |
| ribonucleoprotein complex biogenesis (GO:0022613) | 3 | 1.97 |
| rhythmic process (GO:0048511) | 2 | 1.95 |
| amide biosynthetic process (GO:0043604) | 6 | 1.91 |
| anatomical structure formation involved in morphogenesis (GO:0048646) | 2 | 1.66 |
| translation (GO:0006412) | 5 | 1.56 |
| peptide biosynthetic process (GO:0043043) | 5 | 1.51 |
| pyrimidine-containing compound metabolic process (GO:0072527) | 2 | 1.50 |
| pyrimidine-containing compound biosynthetic process (GO:0072528) | 2 | 1.50 |
| macromolecule catabolic process (GO:0009057) | 4 | 1.47 |
| sulfur amino acid biosynthetic process (GO:0000097) | 2 | 1.45 |
| small molecule metabolic process (GO:0044281) | 11 | 1.44 |
| sulfur amino acid metabolic process (GO:0000096) | 2 | 1.41 |
| rRNA processing (GO:0006364) | 2 | 1.37 |
| cellular modified amino acid metabolic process (GO:0006575) | 2 | 1.37 |
| rRNA metabolic process (GO:0016072) | 2 | 1.37 |
| threonine metabolic process (GO:0006566) | 1 | 1.31 |
| protein targeting (GO:0006605) | 1 | 1.31 |
| riboflavin metabolic process (GO:0006771) | 1 | 1.31 |
| cell-cell signaling (GO:0007267) | 1 | 1.31 |
| circadian rhythm (GO:0007623) | 1 | 1.31 |
| threonine biosynthetic process (GO:0009088) | 1 | 1.31 |
| riboflavin biosynthetic process (GO:0009231) | 1 | 1.31 |
| induced systemic resistance (GO:0009682) | 1 | 1.31 |
| induced systemic resistance, jasmonic acid mediated signaling pathway (GO:0009864) | 1 | 1.31 |
| xylem development (GO:0010089) | 1 | 1.31 |
| phytosteroid metabolic process (GO:0016128) | 1 | 1.31 |
| phytosteroid biosynthetic process (GO:0016129) | 1 | 1.31 |
| brassinosteroid metabolic process (GO:0016131) | 1 | 1.31 |
| brassinosteroid biosynthetic process (GO:0016132) | 1 | 1.31 |
| second-messenger-mediated signaling (GO:0019932) | 1 | 1.31 |
| positive regulation of cellular amide metabolic process (GO:0034250) | 1 | 1.31 |
| flavin-containing compound biosynthetic process (GO:0042727) | 1 | 1.31 |
| positive regulation of translation (GO:0045727) | 1 | 1.31 |
| hydrogen sulfide metabolic process (GO:0070813) | 1 | 1.31 |
| hydrogen sulfide biosynthetic process (GO:0070814) | 1 | 1.31 |
| cellular response to organonitrogen compound (GO:0071417) | 1 | 1.31 |

*P values of all GO terms are lower than 0.05. Conversely, −log 10 (P value) values of all GO terms are greater than 1.3010, that is, the greater -log 10(P value) value, the better significance.
