# Supplementary material for: Comparative Transcriptome Analysis Reveals the Transcriptional Alterations in Growth- and Development-Related Genes in Sweet Potato Plants Infected and Non-Infected by SPFMV, SPV2, and SPVG
Source: Int J Mol Sci. 2019 Feb 26;20(5):1012. doi: 10.3390/ijms20051012 (PMC6429102; doi:10.3390/ijms20051012)
Supplement: Supplementary file 1 [file ijms-20-01012-s001.zip › Supplementary Files/Table S3.doc]

Table S3 Overall GO classification of differentially down-regulated genes of VFSP/VCSP leaves

| Gene Ontology term | Number of genes | -log_10_(P value)* |
| --- | --- | --- |
| **Cell component** |  |  |
| ribosome （GO:0005840） | 52 | 11.57 |
| intracellular ribonucleoprotein complex （GO:0030529） | 66 | 8.00 |
| ribonucleoprotein complex （GO:1990904） | 66 | 7.85 |
| mitochondrial part （GO:0044429） | 34 | 4.35 |
| mitochondrial envelope （GO:0005740） | 26 | 3.85 |
| macromolecular complex （GO:0032991） | 106 | 3.77 |
| mitochondrial membrane （GO:0031966） | 25 | 3.70 |
| mitochondrial inner membrane （GO:0005743） | 18 | 2.81 |
| nucleus （GO:0005634） | 221 | 2.19 |
| proteasome core complex, beta-subunit complex （GO:0019774） | 5 | 2.12 |
| proteasome core complex （GO:0005839） | 8 | 2.09 |
| mitochondrial intermembrane space （GO:0005758） | 4 | 2.00 |
| intracellular （GO:0005622） | 605 | 1.76 |
| intracellular part （GO:0044424） | 605 | 1.76 |
| Golgi stack （GO:0005795） | 6 | 1.71 |
| Golgi cisterna （GO:0031985） | 6 | 1.71 |
| Golgi cisterna membrane （GO:0032580） | 6 | 1.71 |
| respiratory chain （GO:0070469） | 7 | 1.69 |
| organelle envelope lumen （GO:0031970） | 4 | 1.61 |
| endoplasmic reticulum lumen （GO:0005788） | 8 | 1.53 |
| organelle inner membrane （GO:0019866） | 23 | 1.52 |
| cell （GO:0005623） | 675 | 1.41 |
| cell part （GO:0044464） | 675 | 1.41 |
| non-membrane-bounded organelle （GO:0043228） | 81 | 1.39 |
| intracellular non-membrane-bounded organelle （GO:0043232） | 81 | 1.39 |
| peroxisome （GO:0005777） | 18 | 1.33 |
| microbody (GO:0042579) | 18 | 1.33 |
| **Molecular function** |  | 0.00 |
| chitin binding （GO:0008061） | 7 | 3.72 |
| chitinase activity （GO:0004568） | 7 | 2.72 |
| threonine-type endopeptidase activity （GO:0004298） | 8 | 2.03 |
| threonine-type peptidase activity （GO:0070003） | 8 | 2.03 |
| ammonia-lyase activity （GO:0016841） | 4 | 1.97 |
| NADH dehydrogenase activity （GO:0003954） | 3 | 1.93 |
| NADH dehydrogenase (ubiquinone) activity （GO:0008137） | 3 | 1.93 |
| phenylalanine ammonia-lyase activity （GO:0045548） | 3 | 1.93 |
| NADH dehydrogenase (quinone) activity （GO:0050136） | 3 | 1.93 |
| galactosyltransferase activity （GO:0008378） | 6 | 1.92 |
| aldehyde-lyase activity （GO:0016832） | 5 | 1.75 |
| carbon-carbon lyase activity （GO:0016830） | 19 | 1.70 |
| carboxy-lyase activity （GO:0016831） | 14 | 1.68 |
| endopeptidase activity （GO:0004175） | 10 | 1.67 |
| aldehyde dehydrogenase (NAD) activity （GO:0004029） | 6 | 1.67 |
| O-acyltransferase activity （GO:0008374） | 6 | 1.67 |
| dioxygenase activity （GO:0051213） | 16 | 1.66 |
| transaminase activity （GO:0008483） | 9 | 1.50 |
| oxidoreductase activity, acting on the aldehyde or oxo group of donors, NAD or NADP as acceptor （GO:0016620） | 9 | 1.50 |
| transferase activity, transferring nitrogenous groups （GO:0016769） | 9 | 1.50 |
| lyase activity （GO:0016829） | 39 | 1.47 |
| asparagine synthase (glutamine-hydrolyzing) activity （GO:0004066） | 3 | 1.41 |
| pyruvate decarboxylase activity （GO:0004737） | 3 | 1.41 |
| steroid binding （GO:0005496） | 3 | 1.41 |
| 4-coumarate-CoA ligase activity （GO:0016207） | 3 | 1.41 |
| **Biological process** |  |  |
| regulation of RNA metabolic process (GO:0051252) | 112 | 4.28 |
| regulation of transcription, DNA-templated (GO:0006355) | 111 | 4.22 |
| regulation of nucleic acid-templated transcription (GO:1903506) | 111 | 4.22 |
| regulation of RNA biosynthetic process (GO:2001141) | 111 | 4.22 |
| transcription, DNA-templated (GO:0006351) | 111 | 4.17 |
| nucleic acid-templated transcription (GO:0097659) | 111 | 4.17 |
| regulation of nucleobase-containing compound metabolic process (GO:0019219) | 112 | 4.05 |
| RNA biosynthetic process (GO:0032774) | 111 | 4.00 |
| regulation of nitrogen compound metabolic process (GO:0051171) | 119 | 4.00 |
| regulation of macromolecule biosynthetic process (GO:0010556) | 117 | 3.82 |
| regulation of cellular macromolecule biosynthetic process (GO:2000112) | 117 | 3.82 |
| regulation of biosynthetic process (GO:0009889) | 118 | 3.64 |
| regulation of cellular biosynthetic process (GO:0031326) | 118 | 3.64 |
| regulation of gene expression (GO:0010468) | 123 | 3.60 |
| regulation of cellular metabolic process (GO:0031323) | 121 | 3.54 |
| nucleobase-containing compound biosynthetic process (GO:0034654) | 124 | 3.51 |
| regulation of primary metabolic process (GO:0080090) | 119 | 3.49 |
| regulation of macromolecule metabolic process (GO:0060255) | 124 | 3.46 |
| organic cyclic compound biosynthetic process (GO:1901362) | 150 | 3.40 |
| organonitrogen compound catabolic process (GO:1901565) | 15 | 3.39 |
| aromatic compound biosynthetic process (GO:0019438) | 140 | 3.30 |
| tyrosine metabolic process (GO:0006570) | 5 | 3.29 |
| RNA metabolic process (GO:0016070) | 138 | 3.24 |
| aminoglycan metabolic process (GO:0006022) | 7 | 3.24 |
| aminoglycan catabolic process (GO:0006026) | 7 | 3.24 |
| chitin metabolic process (GO:0006030) | 7 | 3.24 |
| chitin catabolic process （GO:0006032） | 7 | 3.24 |
| amino sugar catabolic process （GO:0046348） | 7 | 3.24 |
| glucosamine-containing compound metabolic process （GO:1901071） | 7 | 3.24 |
| glucosamine-containing compound catabolic process （GO:1901072） | 7 | 3.24 |
| carbohydrate derivative catabolic process（GO:1901136） | 7 | 3.24 |
| regulation of metabolic process （GO:0019222） | 126 | 3.19 |
| response to stress (GO:0006950) | 93 | 3.19 |
| response to salt stress (GO:0009651) | 12 | 3.07 |
| organic substance metabolic process (GO:0071704) | 424 | 2.94 |
| response to osmotic stress (GO:0006970) | 13 | 2.82 |
| phenylpropanoid biosynthetic process (GO:0009699) | 9 | 2.80 |
| gene expression (GO:0010467) | 158 | 2.66 |
| L-phenylalanine metabolic process (GO:0006558) | 4 | 2.63 |
| erythrose 4-phosphate/phosphoenolpyruvate family amino acid metabolic process (GO:1902221) | 4 | 2.63 |
| biological regulation (GO:0065007) | 179 | 2.63 |
| regulation of biological process（GO:0050789） | 171 | 2.63 |
| organic cyclic compound metabolic process（GO:1901360） | 207 | 2.60 |
| cellular aromatic compound metabolic process（GO:0006725） | 198 | 2.52 |
| phenylpropanoid metabolic process （GO:0009698） | 14 | 2.47 |
| heterocycle biosynthetic process （GO:0018130） | 134 | 2.45 |
| cellular amino acid metabolic process （GO:0006520） | 32 | 2.43 |
| alpha-amino acid metabolic process （GO:1901605） | 30 | 2.41 |
| nitrogen compound metabolic process （GO:0006807） | 240 | 2.41 |
| primary metabolic process （GO:0044238） | 379 | 2.36 |
| defense response （GO:0006952） | 54 | 2.36 |
| carboxylic acid metabolic process （GO:0019752） | 73 | 2.36 |
| regulation of cellular process （GO:0050794） | 152 | 2.35 |
| secondary metabolite biosynthetic process （GO:0044550） | 9 | 2.30 |
| positive regulation of immune system process （GO:0002684） | 6 | 2.29 |
| positive regulation of innate immune response （GO:0045089） | 6 | 2.29 |
| positive regulation of immune response （GO:0050778） | 6 | 2.29 |
| monocarboxylic acid metabolic process （GO:0032787） | 40 | 2.07 |
| response to stimulus（GO:0050896） | 138 | 2.05 |
| activation of innate immune response （GO:0002218） | 4 | 2.02 |
| activation of immune response （GO:0002253） | 4 | 2.02 |
| benzene-containing compound metabolic process （GO:0042537） | 4 | 2.02 |
| regulation of response to salt stress （GO:1901000） | 4 | 2.02 |
| aromatic amino acid family catabolic process （GO:0009074） | 3 | 1.97 |
| aromatic amino acid family biosynthetic process, prephenate pathway （GO:0009095） | 3 | 1.97 |
| cinnamic acid biosynthetic process （GO:0009800） | 3 | 1.97 |
| cinnamic acid metabolic process （GO:0009803） | 3 | 1.97 |
| auxin homeostasis （GO:0010252） | 3 | 1.97 |
| aldonic acid metabolic process （GO:0019520） | 3 | 1.97 |
| cellular response to osmotic stress （GO:0071470） | 3 | 1.97 |
| cellular aldehyde metabolic process （GO:0006081） | 11 | 1.94 |
| nucleobase-containing compound metabolic process （GO:0006139） | 170 | 1.94 |
| amino sugar metabolic process （GO:0006040） | 7 | 1.92 |
| cellular nitrogen compound biosynthetic process （GO:0044271） | 151 | 1.92 |
| response to bacterium （GO:0009617） | 14 | 1.91 |
| nucleic acid metabolic process （GO:0090304） | 145 | 1.90 |
| ribose phosphate metabolic process （GO:0019693） | 21 | 1.86 |
| immune response （GO:0006955） | 15 | 1.84 |
| innate immune response（GO:0045087） | 15 | 1.84 |
| lateral root development （GO:0048527） | 5 | 1.80 |
| post-embryonic root development （GO:0048528） | 5 | 1.80 |
| multi-organism process （GO:0051704） | 30 | 1.80 |
| flavonoid metabolic process （GO:0009812） | 9 | 1.74 |
| immune system process （GO:0002376） | 15 | 1.73 |
| methionine metabolic process （GO:0006555） | 6 | 1.73 |
| positive regulation of defense response （GO:0031349） | 6 | 1.73 |
| regulation of molecular function （GO:0065009） | 6 | 1.73 |
| aromatic amino acid family metabolic process （GO:0009072） | 8 | 1.72 |
| heterocycle metabolic process（GO:0046483） | 186 | 1.69 |
| response to inorganic substance （GO:0010035） | 14 | 1.68 |
| secondary metabolic process （GO:0019748） | 14 | 1.68 |
| aspartate family amino acid metabolic process （GO:0009066） | 11 | 1.66 |
| regulation of response to stimulus（GO:0048583） | 19 | 1.66 |
| oxoacid metabolic process （GO:0043436） | 73 | 1.65 |
| response to abiotic stimulus （GO:0009628） | 34 | 1.64 |
| response to UV （GO:0009411） | 4 | 1.62 |
| post-embryonic root morphogenesis （GO:0010101） | 4 | 1.62 |
| lateral root morphogenesis （GO:0010102） | 4 | 1.62 |
| lateral root formation （GO:0010311） | 4 | 1.62 |
| regulation of response to osmotic stress （GO:0047484） | 4 | 1.62 |
| cellular amino acid biosynthetic process （GO:0008652） | 24 | 1.62 |
| regulation of defense response （GO:0031347） | 10 | 1.62 |
| organic acid metabolic process (GO:0006082) | 73 | 1.61 |
| regulation of response to stress (GO:0080134) | 12 | 1.59 |
| alpha-amino acid biosynthetic process (GO:1901607) | 22 | 1.59 |
| organic acid catabolic process (GO:0016054) | 9 | 1.58 |
| carboxylic acid catabolic process (GO:0046395) | 9 | 1.58 |
| flavonoid biosynthetic process (GO:0009813) | 8 | 1.55 |
| post-embryonic plant organ development (GO:0090696) | 5 | 1.54 |
| regulation of innate immune response (GO:0045088) | 7 | 1.53 |
| regulation of immune response (GO:0050776) | 7 | 1.53 |
| cellular amino acid catabolic process (GO:0009063) | 6 | 1.52 |
| leaf senescence (GO:0010150) | 6 | 1.52 |
| plant organ senescence (GO:0090693) | 6 | 1.52 |
| alpha-amino acid catabolic process (GO:1901606) | 6 | 1.52 |
| divalent metal ion transport (GO:0070838) | 10 | 1.49 |
| divalent inorganic cation transport (GO:0072511) | 10 | 1.49 |
| small molecule metabolic process (GO:0044281) | 95 | 1.48 |
| response to external biotic stimulus (GO:0043207) | 20 | 1.48 |
| response to other organism (GO:0051707) | 20 | 1.48 |
| response to oxygen-containing compound (GO:1901700) | 33 | 1.48 |
| carbohydrate derivative metabolic process (GO:1901135) | 38 | 1.47 |
| response to molecule of bacterial origin （GO:0002237） | 3 | 1.45 |
| asparagine metabolic process （GO:0006528） | 3 | 1.45 |
| asparagine biosynthetic process （GO:0006529） | 3 | 1.45 |
| zinc II ion transport （GO:0006829） | 3 | 1.45 |
| response to hydrogen peroxide（GO:0042542） | 3 | 1.45 |
| negative regulation of molecular function （GO:0044092） | 3 | 1.45 |
| L-asparagine biosynthetic process （GO:0070981） | 3 | 1.45 |
| L-asparagine metabolic process （GO:0070982） | 3 | 1.45 |
| ribonucleotide biosynthetic process （GO:0009260） | 9 | 1.45 |
| ribose phosphate biosynthetic process （GO:0046390） | 9 | 1.45 |
| positive regulation of response to stimulus （GO:0048584） | 9 | 1.45 |
| cellular macromolecule biosynthetic process （GO:0034645） | 137 | 1.44 |
| cellular nitrogen compound metabolic process （GO:0034641） | 203 | 1.41 |
| sulfur amino acid metabolic process （GO:0000096） | 8 | 1.40 |
| response to biotic stimulus （GO:0009607） | 21 | 1.39 |
| small molecule catabolic process （GO:0044282） | 10 | 1.37 |
| regulation of immune system process （GO:0002682） | 7 | 1.37 |
| purine nucleotide biosynthetic process （GO:0006164） | 7 | 1.37 |
| purine ribonucleotide biosynthetic process （GO:0009152） | 7 | 1.37 |
| programmed cell death （GO:0012501） | 7 | 1.37 |
| defense response to other organism （GO:0098542） | 15 | 1.36 |
| biosynthetic process （GO:0009058） | 234 | 1.35 |
| isoleucine metabolic process （GO:0006549） | 4 | 1.34 |
| polyamine metabolic process （GO:0006595） | 4 | 1.34 |
| polyamine biosynthetic process （GO:0006596） | 4 | 1.34 |
| isoleucine biosynthetic process （GO:0009097） | 4 | 1.34 |
| post-embryonic plant organ morphogenesis （GO:0090697） | 4 | 1.34 |
| transition metal ion transport （GO:0000041） | 5 | 1.32 |
| methionine biosynthetic process （GO:0009086） | 5 | 1.32 |
| lignin biosynthetic process （GO:0009809） | 5 | 1.32 |
| cellular biosynthetic process （GO:0044249） | 220 | 1.32 |
| ribonucleotide metabolic process （GO:0009259） | 17 | 1.31 |
| organic substance biosynthetic process （GO:1901576） | 231 | 1.31 |

*P values of all GO terms are lower than 0.05. Conversely, −log 10 (P value) values of all GO terms are greater than 1.3010, that is, the greater -log 10(P value) value, the better significance.
