# Supplementary material for: Comparative Transcriptome Analysis Reveals the Transcriptional Alterations in Growth- and Development-Related Genes in Sweet Potato Plants Infected and Non-Infected by SPFMV, SPV2, and SPVG
Source: Int J Mol Sci. 2019 Feb 26;20(5):1012. doi: 10.3390/ijms20051012 (PMC6429102; doi:10.3390/ijms20051012)
Supplement: Supplementary file 1 [file ijms-20-01012-s001.zip › Supplementary Files/Table S4.doc]

Table S4 Overall GO classification of differentially down-regulated genes of VCSP and VFSP storage roots

| Gene ontology term | Number of genes | -log_10_(P value)* |
| --- | --- | --- |
| **Cell component** |  |  |
| extracellular region (GO:0005576) | 10 | 2.17 |
| **Molecular function** |  |  |
| hydrolase activity, hydrolyzing O-glycosyl compounds (GO:0004553) | 8 | 3.14 |
| aldo-keto reductase (NADP) activity (GO:0004033) | 2 | 2.88 |
| hydrolase activity, acting on glycosyl bonds (GO:0016798) | 8 | 2.62 |
| glutamate-ammonia ligase activity (GO:0004356) | 2 | 2.59 |
| ammonia ligase activity (GO:0016211) | 2 | 2.59 |
| acid-ammonia (or amide) ligase activity (GO:0016880) | 2 | 2.59 |
| glucosidase activity (GO:0015926) | 4 | 2.42 |
| carbon-oxygen lyase activity, acting on polysaccharides (GO:0016837) | 2 | 2.37 |
| pectate lyase activity (GO:0030570) | 2 | 2.37 |
| beta-fructofuranosidase activity (GO:0004564) | 2 | 2.06 |
| sucrose alpha-glucosidase activity (GO:0004575) | 2 | 2.06 |
| carbon-oxygen lyase activity (GO:0016835) | 4 | 1.94 |
| alpha-glucosidase activity (GO:0090599) | 2 | 1.84 |
| galactosyltransferase activity (GO:0008378) | 2 | 1.75 |
| lyase activity (GO:0016829) | 7 | 1.70 |
| cellulase activity (GO:0008810) | 2 | 1.59 |
| carbohydrate binding (GO:0030246) | 3 | 1.47 |
| transferase activity, transferring alkyl or aryl (other than methyl) groups (GO:0016765) | 3 | 1.43 |
| carboxylic ester hydrolase activity (GO:0052689) | 3 | 1.43 |
| glucan endo-1,3-beta-D-glucosidase activity (GO:0042973) | 2 | 1.41 |
| carboxy-lyase activity (GO:0016831) | 3 | 1.40 |
| pectinesterase activity (GO:0030599) | 2 | 1.36 |
| aspartyl esterase activity (GO:0045330) | 2 | 1.36 |
| **Biological process** |  |  |
| polysaccharide catabolic process (GO:0000272) | 7 | 3.72 |
| pectin catabolic process (GO:0045490) | 4 | 3.00 |
| macromolecule catabolic process (GO:0009057) | 7 | 2.97 |
| nitrogen fixation (GO:0009399) | 2 | 2.84 |
| beta-glucan catabolic process (GO:0051275) | 3 | 2.59 |
| carbohydrate metabolic process (GO:0005975) | 12 | 2.53 |
| carbohydrate catabolic process (GO:0016052) | 7 | 2.53 |
| glucan catabolic process (GO:0009251) | 3 | 2.41 |
| cellular polysaccharide catabolic process (GO:0044247) | 3 | 2.41 |
| cellular carbohydrate catabolic process (GO:0044275) | 3 | 2.32 |
| spermidine metabolic process (GO:0008216) | 2 | 2.16 |
| spermidine biosynthetic process (GO:0008295) | 2 | 2.16 |
| polysaccharide metabolic process (GO:0005976) | 7 | 2.12 |
| pectin metabolic process (GO:0045488) | 4 | 2.12 |
| galacturonan metabolic process (GO:0010393) | 4 | 2.07 |
| polyamine metabolic process (GO:0006595) | 2 | 2.02 |
| polyamine biosynthetic process (GO:0006596) | 2 | 2.02 |
| organic substance catabolic process (GO:1901575) | 10 | 2.00 |
| catabolic process (GO:0009056) | 11 | 1.87 |
| iron ion homeostasis (GO:0055072) | 2 | 1.80 |
| beta-glucan metabolic process (GO:0051273) | 3 | 1.72 |
| cell wall organization (GO:0071555) | 7 | 1.59 |
| cellulose catabolic process (GO:0030245) | 2 | 1.56 |
| external encapsulating structure organization (GO:0045229) | 7 | 1.55 |
| nucleoside metabolic process (GO:0009116) | 2 | 1.49 |
| ribonucleoside metabolic process (GO:0009119) | 2 | 1.49 |
| nucleoside biosynthetic process (GO:0009163) | 2 | 1.49 |
| ribonucleoside biosynthetic process (GO:0042455) | 2 | 1.49 |
| transition metal ion homeostasis (GO:0055076) | 2 | 1.49 |
| ammonium ion metabolic process (GO:0097164) | 2 | 1.49 |
| cell wall organization or biogenesis (GO:0071554) | 7 | 1.47 |
| amine biosynthetic process (GO:0009309) | 2 | 1.43 |
| cellular biogenic amine biosynthetic process (GO:0042401) | 2 | 1.43 |
| cellular biogenic amine metabolic process (GO:0006576) | 2 | 1.37 |
| cellular amine metabolic process (GO:0044106) | 2 | 1.37 |
| metal ion homeostasis (GO:0055065) | 2 | 1.37 |
| cation homeostasis (GO:0055080) | 2 | 1.37 |
| inorganic ion homeostasis (GO:0098771) | 2 | 1.32 |
| glycosyl compound metabolic process (GO:1901657) | 2 | 1.32 |
| glycosyl compound biosynthetic process (GO:1901659) | 2 | 1.32 |

*P values of all GO terms are lower than 0.05. Conversely, −log 10 (P value) values of all GO terms are greater than 1.3010, that is, the greater -log 10(P value) value, the better significance.
